# Withaferin A activates TRIM16 for its anti-cancer activity in melanoma

**DOI:** 10.1038/s41598-020-76722-x

**Published:** 2020-11-12

**Authors:** Zsuzsanna Nagy, Belamy B. Cheung, Wing Tsang, Owen Tan, Mika Herath, Olivia C. Ciampa, Fatima Shadma, Daniel R. Carter, Glenn M. Marshall

**Affiliations:** 1 Children’s Cancer Institute Australia for Medical Research, Lowy Cancer Research Centre, UNSW Sydney, PO Box 81, Randwick, NSW 2031 Australia; 2 School of Women’s and Children’s Health, UNSW Sydney, Randwick, NSW 2031 Australia; 3 Academy of Medical Sciences, Zhengzhou University, Henan, China; 4 School of Biomedical Engineering, University of Technology, Sydney, NSW 2007 Australia; 5 Kids Cancer Centre, Sydney Children’s Hospital, Level 1, South Wing, High Street, Randwick, NSW 2031 Australia

**Keywords:** Cancer, Drug discovery, Molecular biology, Medical research, Oncology

## Abstract

Although selective BRAF inhibitors and novel immunotherapies have improved short-term treatment responses in metastatic melanoma patients, acquired resistance to these therapeutics still represent a major challenge in clinical practice. In this study, we evaluated the efficacy of Withaferin A (WFA), derived from the medicinal plant *Withania Somnifera*, as a novel therapeutic agent for the treatment of melanoma. WFA showed selective toxicity to melanoma cells compared to non-malignant cells. WFA induced apoptosis, significantly reduced cell proliferation and inhibited migration of melanoma cells. We identified that repression of the tumour suppressor TRIM16 diminished WFA cytotoxicity, suggesting that TRIM16 was in part responsible for the cytotoxic effects of WFA in melanoma cells. Together our data indicates that WFA has potent cytopathic effects on melanoma cells through TRIM16, suggesting a potential therapeutic application of WFA in the disease.

## Introduction

Malignant melanoma is the deadliest cutaneous neoplasm. Acquired drug resistance frequently develops after a period of objective tumor response, justifying the need for novel therapies^1,2^. Withaferin A (WFA), a steroidal lactone extracted from *Withania somnifera*, has been described as a potential anti-cancer drug both in vivo and in vitro^3,4^ through its diverse anti-tumour properties and low cytotoxicity to non-malignant cells. WFA is a promising therapeutic agent for a broad range of cancers, however its mechanism of action is understudied. Recent research has demonstrated a number of possible mechanisms of action for WFA, such as direct inhibition of the intermediate filament vimentin^5,6^. Inhibition of vimentin reduces formation of metastasis in pre-clinical models of breast cancer, osteosarcoma and lung cancer, melanoma and hepatocellular carcinoma^7–11^. We have reported^12^ that the expression of *TRIM16* (tripartite motif 16) is significantly reduced during normal skin transition to squamous cell carcinoma. We have also shown that TRIM16 acts as a tumour suppressor and reduces cell motility via down-regulation of vimentin expression^12^. We have also shown that high TRIM16 protein expression is associated with favourable outcome in melanoma patients with stage III disease^13^. Moreover, suppression of TRIM16 expression increased migration of normal human epidermal melanocytes, while overexpression of TRIM16 reduced melanoma cell migration and proliferation^13^. The above detailed evidence suggested that increased *TRIM16* expression is a potential molecular target for the treatment of melanoma.

## Results

### WFA has increased cytotoxicity for melanoma cells compared with fibroblast cell lines

**Figure 1:**
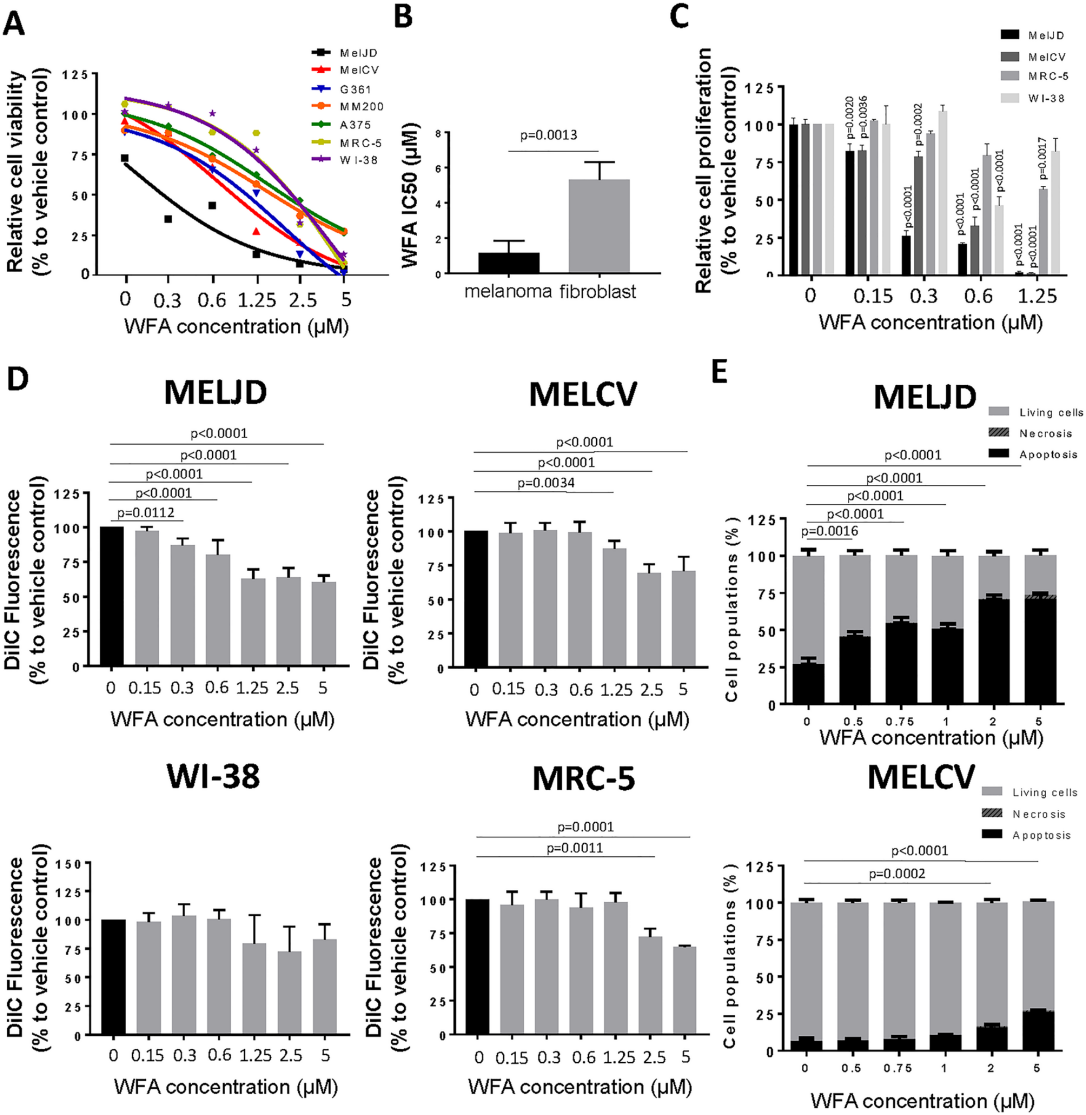
WFA has selective toxicity to melanoma cells compared with fibroblast cell lines. (**A**), A panel of melanoma (MelJD, MelCV, G361, A375 and MM200) cells and normal fibroblasts (WI-38, MRC-5) were treated with increasing concentrations (0–5 μM) of Withaferin A (WFA) for 48 h and cell viability was measured using the Alamar Blue assay. (**B**), Average IC50 for WFA-treated melanoma cell lines and normal fibroblasts. Melanoma cell lines (MelJD and MelCV) and normal fibroblasts (WI-38, and MRC-5) were treated with increasing concentrations (0–5 μM) of WFA for 48 h, followed by (**C**), BrdU cell proliferation assay (**D**), MITOPROBE DILC_1_(5) measurements. (**E**), Flow cytometry results of MelJD and MelCV cells treated with increasing concentrations of WFA, then stained with Annexin-V-FITC/7AAD. Results are mean ± SEM, data was normalized to vehicle control treatment group.

**Figure 2:**
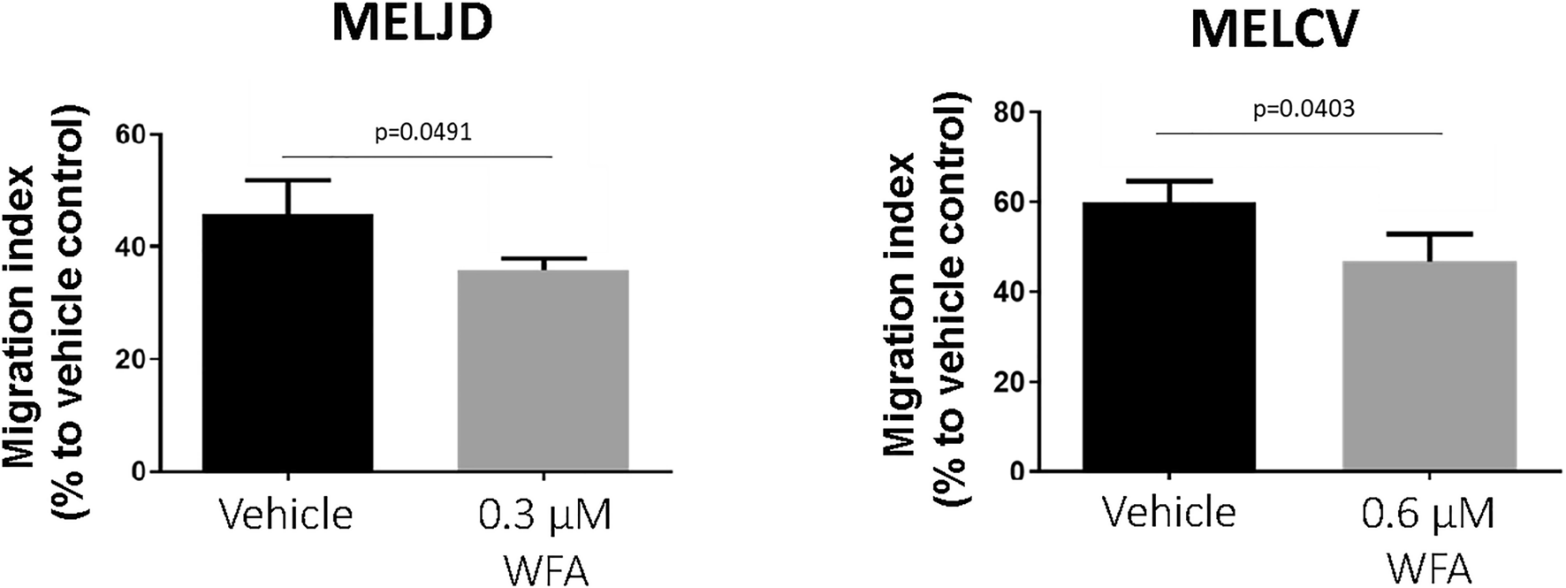
WFA treatment decreases melanoma cell migration. MelJD and MelCV cells treated with indicated concentrations of WFA for 18 h. Results are mean ± SEM, data was normalized to vehicle control treatment group.

To determine whether WFA had selective cytotoxicity to melanoma cells over non-malignant cells, WFA was screened at a range (0–5 μM) of concentrations for its effects on cell viability (Fig. 1A) against five melanoma cell lines (MelCV, MelJD, G3601, A375 and MM200) compared with normal human lung fibroblasts (MRC-5 and WI-38). WFA demonstrated marked single agent activity against three of the five melanoma cell lines (MelJD, MelCV, G361) and reduced toxicity toward normal fibroblasts (Fig. 1A). Comparison of overall IC50s of the melanoma cell lines and fibroblast cells (Fig. 1B) showed a significant increase in cytotoxicity for melanoma cells. WFA also exerted a concentration-dependent anti-proliferative effect (Fig. 1C) measured by the BrdU cell proliferation assay in the case of MelJD and MelCV melanoma cells when compared to fibroblasts. Anti-proliferative actions developed at much lower WFA concentrations (Fig. 1C). To determine the effect of WFA treatment on cell death, mitochondrial membrane potential (one of the earliest markers of apoptosis) was measured using a *MITOPROBE DILC*_*1*_*(5)* Assay. WFA treatment significantly decreased the mitochodrial membrane potential of MelJD and MelCV cells in a concentration-dependent manner (Fig. 1D), while WI-38 and MRC-5 normal fibroblasts were less sensitive to the treatment and very marginal. In order to further investigate early apoptotic and necrotic processes during treatment, we further assessed the effects of WFA on melanoma cells by measuring Annexin-V positivity (Fig. 1E and Supplementary Fig. 1) of MelJD and MelCV cells treated with increasing WFA concentrations (0–5 µM). Importantly, we found that in line with our cell viability data, WFA induced apoptotic and necrotic events in MelJD and MelCV cells in a concentration-dependent manner (Fig. 1E). To investigate the effect of WFA treatment on cell migration, we examined the motility of MelJD and MelCV cells in a trans-well assay. As shown in Fig. 2, MelJD and MelCV cells treated with WFA demonstrated slower cell migration compared to the vehicle treated control cells.

### WFA induces TRIM16 protein expression in melanoma cells

**Figure 3:**
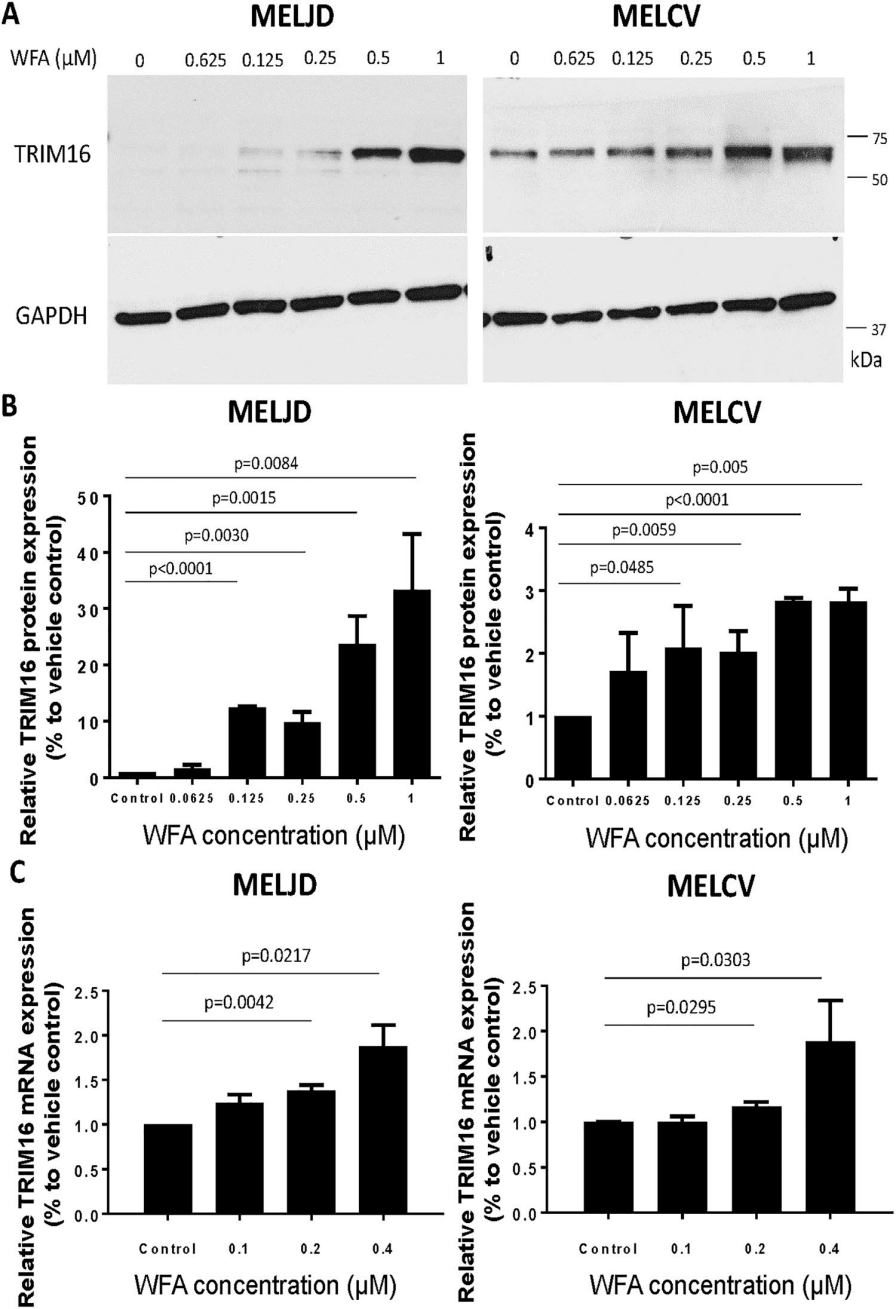
WFA treatment induces TRIM16 protein expression. (**A**), Western blot images of TRIM16 and GAPDH protein expression in MelJD and MelCV cells treated with WFA at increasing concentrations. The full-length blots are presented in the Supplementary Fig. 3A. (**B**), The level of TRIM16 protein expression measured by densitometry quantification. (**C**), RT-qPCR analysis of TRIM16 mRNA expression in MelJD and MelCV cells treated with WFA at increasing concentrations. Results are mean ± SEM, data was normalized to vehicle control treatment group.

To determine whether TRIM16 is responsible for the anti-cancer effects of WFA in melanoma cells, MelJD and MelCV cells were treated with increasing concentrations of WFA and changes in TRIM16 protein and mRNA expression was analyzed by western blot and RT-qPCR. WFA treatment increased TRIM16 protein expression in both MelJD and MelCV cells in a dose-dependent manner (Fig. 3A, B). TRIM16 mRNA expression levels also increased (Fig. 3C) in both MelJD and MelCV cells during WFA treatment, suggesting that WFA induces TRIM16 expression at a transcriptional level.

### WFA is partially dependent on TRIM16 for its cytotoxic activity

**Figure 4:**
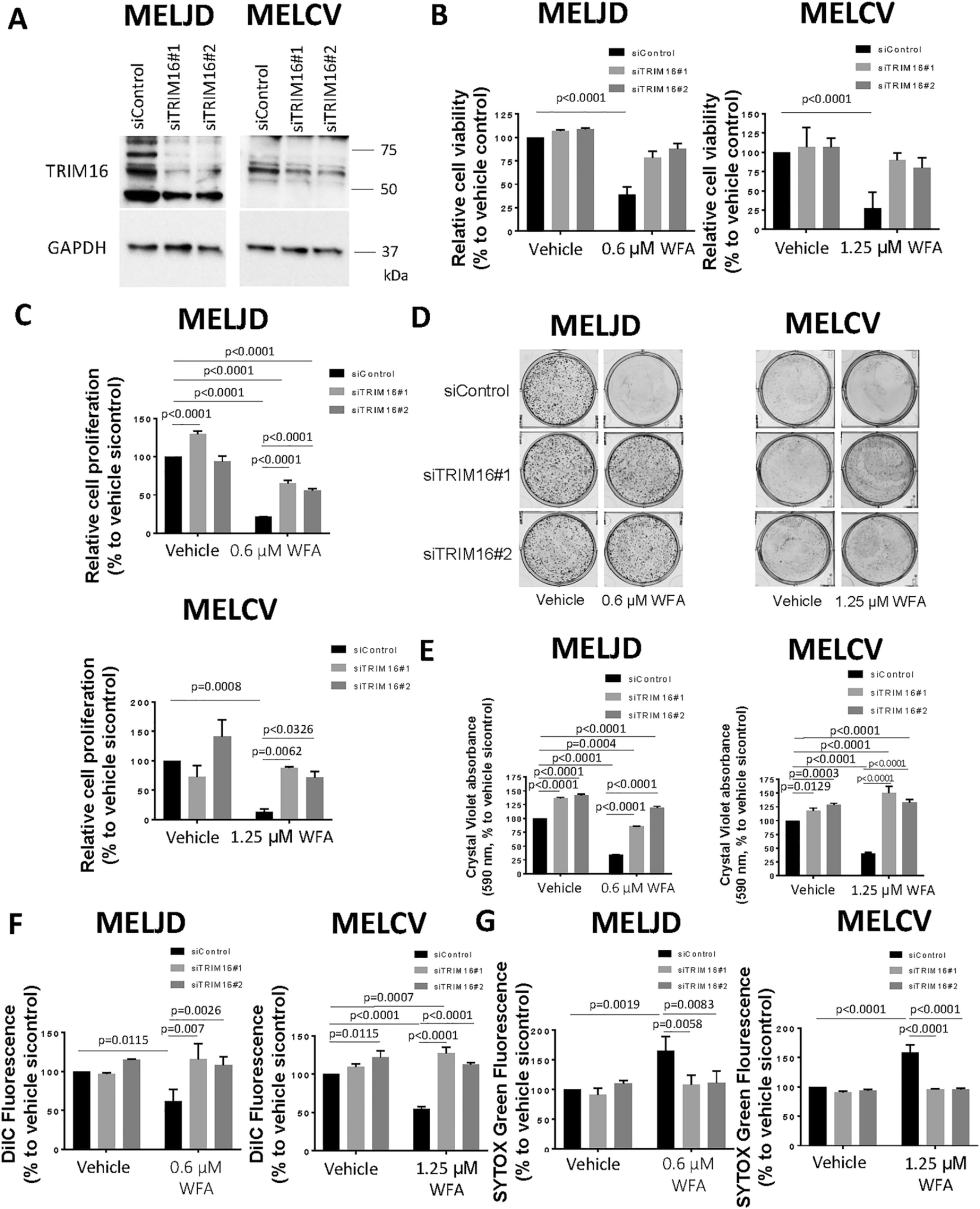
Knock-down of TRIM16 protects melanoma cells against WFA. (**A**), Western blot analysis of TRIM16 expression in MelJD and MelCV cells following TRIM16 knock-down. GAPDH was used as internal control. The full-length blots are presented in the Supplementary Fig. 3B. MelJD and MelCV cells were transfected with control siRNA (siControl) or TRIM16 siRNAs (siTRIM16) for 24 h, then treated with WFA for additional 48 h followed by (**B**), Alamar Blue cell viability and (**C**), and BrdU cell proliferation measurements. Results are mean ± SEM, differences in cell viability and proliferation were compared to the vehicle (DMSO) treated siRNA control cells. (**D**), MelJD and MelCV cells expressing TRIM16 siRNAs (siTRIM16) or control siRNA (siControl) were seeded for colony formation assay and treated with indictaed concentrations of WFA for 72 h then allowed for colonies to form for 10 days, followed by crystal violet staining. (**E**), Quantification of colony formation assay based on crystal violet absorbance (590 nm). Differences in colony formation were compared to the vehicle treated control siRNA. MelJD and MelCV cells were transfected with control siRNA (siControl) or TRIM16 siRNAs (siTRIM16) for 24 h, then treated with WFA for additional 48 h followed by (**F,**) MITOPROBE DILC_1_(5) and (**G**), SYTOX Green measurements. Results are mean ± SEM, differences in cell viability and proliferation were compared to the vehicle control siRNA control cells.

To evaluate whether induction of TRIM16 expression was essential for WFA to exert its effect on melanoma cell survival, we silenced TRIM16 gene expression using two TRIM16-specific siRNAs (Fig. 4A and Supplementary Fig. 2) in MelJD and MelCV melanoma cells, followed by treatment with WFA. As expected, WFA treatment significantly reduced cell viability of both MelJD and MelCV cells (Fig. 4B), when compared to DMSO treated siControl (Vehicle) cells. Conversely, we found that knockdown of TRIM16 using TRIM16-specific siRNAs blocked the reduction of cell viability induced by WFA treatment in both MelJD and MelCV cells (Fig. 4B) when compared to DMSO treated siControl (Vehicle) cells. BrdU cell proliferation (Fig. 4C) and colony formation assays (Fig. 4D, E) also showed that while WFA significantly reduced both short and long-term proliferation of both MelJD and MelCV cells, knockdown of TRIM16 rescued the cells from the effects of WFA. Furthermore, WFA treatment significantly decreased the survival of MelJD and MelCV cells as measured by their mitochodrial membrane potential (Fig. 4F), however, cells transfected with TRIM16 specific siRNAs were less sensitive to WFA treatment. We further assessed the effects of TRIM16 expression on necrotic events (SYTOX Green accumulation assay) induced by WFA. We found that in line with our cell viability data, WFA induced necrosis in WFA treated melanoma cells (Fig. 4G), however, knockdown of TRIM16 rescued the cells from the cytotoxic effects induced by WFA.

## Discussion

Although, kinase inhibitors and immunotherapies using checkpoint inhibitors have greatly improved survival of melanoma patients with advanced disease, treatment failure still represents a major clinical issue^1,2^. Hence, there is an urgent need to introduce novel therapeutics for the treatment of melanoma. The therapeutic effects of WFA in regulating cell survival, migration, angiogenesis, proliferation and metastasis in many types of cancers^14^ has drawn more attention toward WFA as an anti-cancer compound. WFA induces mitochondrial dysfunction as well as apoptosis in leukaemia cells^15^, melanoma cells^16^ and breast cancer cells^17^. WFA was also found to prevent angiogenesis by binding to vimentin filaments^5^ and nestin^18^. Although these studies have shown that WFA is an effective anti-cancer compound for a variety of cancers, the molecular mechanism of WFA’s drug action in melanoma is not fully understood. The objective of the present study was to further investigate the anti-cancer potential of WFA against human melanoma and to decipher the molecular mechanisms involved.

Although the effect of WFA on melanoma cell viability^16^ and proliferation^19,20^ has been investigated before, in this study, we conducted a thorough investigation using a wide range of melanoma cell lines, as well as normal fibroblasts to investigate the therapeutic window and concentration range for WFA to be administered without the risk of adverse side-effects. Previous studies have shown that WFA administration inhibits in vivo growth of a variety of tumor xenografts^17,19,21^ including uveal melanoma^19^, as well the sensitizing effect of WFA of B16F1 melanoma cells to radiotherapy^22^. Further investigation using preclinical animal models to test WFA efficacy for melanoma is a natural extension to our current study.

G2/M cell cycle arrest and apoptosis induction following treatment with WFA has been reported for melanoma^16^ and uveal melanoma^19^. In melanoma cell lines, the apoptotic process triggered by WFA involved the mitochondrial pathway and was associated with Bax mitochondrial translocation, cytochrome-c release, transmembrane potential changes, and caspase 9 and caspase 3 activation. WFA cytotoxicity may also require early reactive oxygen species (ROS) production^16^. Consistent with this data, both melanoma cell lines in our study had a concentration-dependent increase in apoptosis. Interestingly, MelJD cells (BRAF*WT*) were more sensitive to the cytotoxic effect of WFA compared to MelCV cells, suggesting that BRAF mutation status might regulate sensitivity to WFA. A more intensive investigation is required to understand the link between WFA sensitivity and BRAF mutational status both in vitro and in vivo.

Here, we have shown that WFA treatment induced *TRIM16* mRNA expression in melanoma cell lines and that TRIM16 was required to induce maximal cytotoxic effect. We found that MelCV melanoma cells were less sensitive to WFA treatment in comparison to MelJD cells. Interestingly, MelCV cells showed a lower basal TRIM16 expression compared to the MelJD cells^13^. We hypothesize that MelCV cells may be intrinsically less sensitive to WFA treatment due to pre-existing lower basal of TRIM16 and that the apoptotic action of TRIM16 may be suppressed by other means in these cells. The mechanism by which TRIM16 expression is lost in melanoma cells is currently unknown. Previous data indicated multiple mechanisms in neuroblastoma, including promoter methylation and reduced protein stability^23^, similar dysregulation may occur in melanoma. We showed that with increasing WFA concentration, the induction of *TRIM16* mRNA expression was only mild, suggesting it is also possible that WFA can act by other regulatory mechanisms such as post-translational modifications that increase the stability or inhibit proteosomal degradation of TRIM16. The latter hypothesis was supported by research that shows WFA can inhibit the proteasome, the site of TRIM16 degradation^12,24^.

WFA treatment effectively reduced the growth of 4T1 mouse mammary tumor xenografts^11^. Growth inhibition was also accompanied by degradation of vimentin^11^. WFA was also found to prevent angiogenesis by binding to the intermediate F-actin and vimetin filaments^5^ and nestin^18^. It has been previously described that TRIM16 bound directly to cytoplasmic vimentin^23^. We hypothesize that the inhibition of this interaction is necessary for the action of WFA on melanoma cells. Stage III melanoma patients with lymph node metastases have high *TRIM16* expression with a longer median survival (59 months) compared to patients with low *TRIM16* expression (16 months)^23^, therefore we investigated the effect of WFA on melanoma cell migration. Consistent with studies conducted in breast cancer cell lines^11^, our migration assay results demonstrated that WFA inhibited melanoma cell migration. Therefore, we suggest that WFA treatment and upregulation of *TRIM16* expression could be a potential method to prevent disease progression and serve as maintenance therapy for Stage II melanoma patients.

Despite promising in vitro data summarized in this article, several steps are still necessary to introduce WFA for prevention and/or therapy of melanoma. Studies on WFA efficacy in vivo would further validate this compound as a candidate for therapeutic development. Second, more intensive investigation is required to understand the link between WFA sensitivity and BRAF mutational status. Last, it is unknown whether TRIM16 reactivation by WFA potentiate another targeted anti-melanoma therapy.

## Materials and methods

### Cell culture

Melanoma cell lines, MelJD, MelCV and MM200 were kindly gifted from Professor Xu Dong Zhang at the University of Newcastle (Newcastle, NSW, Australia). Melanoma cell line G361 and A375 were purchased from ATCC. All melanoma cell lines were maintained in Dulbecco’s modified Eagle’s medium (DMEM) (LIFE TECHNOLOGIES Australia, VIC, Australia) with 5% fetal calf serum (FCS) (LIFE TECHNOLOGIES). MRC-5 and WI-38 normal human fibroblasts were purchased from ATCC and grown in alpha-minimum essential media (MEM) (LIFE TECHNOLOGIES) supplemented with 10% heat inactivated FCS. All cells were freshly thawed from initial seeds, cultured at 37 °C/5% CO_2_ in a humidifier incubator for not more than 2 months.

### siRNA transfection

For siRNA mediated knock-down, the indicated cell lines were transfected with 20 nM of custom designed siRNA duplex oligos (TRIM16 siRNA#1: (5′AGTAATTCACCATGCAGGTTT-3′ and TRIM16 siRNA#2: (5′TCTCCCTCCTGCATTTGTGTT-3′) synthesized by QIAGEN, Australia. Non-targeting pool siRNA was used as siControl and purchased from DHARMACON, Australia. Cells were transfected between 24 to 72 h depending on the experimental requirements. Transfections were performed using Lipofectamine 2000 (LIFE TECHNOLOGIES) following the manufacturer's protocol.

### Pharmacological growth assays

Pharmacological growth inhibition assays were performed using 2 × 10^4^ melanoma cells per well and 8 × 10^3^ fibroblasts cells per well in 96- well microplates. When applicable, cells were transfected with either TRIM16 siRNA#1, TRIM16 siRNA#2 or Control siRNA. 24 h after transfection, serial dilutions of Whitaferin A (WFA) was added to the cells (0–5 µM). Cells were incubated with vehicle control (DMSO) or WFA for 48 h followed by cell viability, cell proliferation, cell death as well as colony formation assays.

### Cell viability assay

The number of viable cells was determined by using the Alamar Blue assay (LIFE TECHNOLOGIES) according to the manufacturer's protocol. Signals were quantitated on a VICTOR MULTILABEL COUNTER (PERKINELMER, Australia) at an excitation wavelength of 560 nm and an emission wavelength of 590 nm.

### Cell proliferation assay

Cell proliferation was measured using BrdU ELISA kit (ROCHE, Australia) according to manufacturer’s instructions. Cells were grown in medium containing 5 μg/ml BrdU for 4 h (MelJD and MelCV) or 6 h (WI-38 and MRC-5) and fixed in 4% paraformaldehyde. DNA was denatured, and cells were permeabilized in 2 N HCl with 0.5% Triton X-100 (SIGMA, Australia) and then blocked with 5% BSA in PBS. Anti-BrdU was added following the manufacturer's protocol. After washing with 5% BSA in PBS, the cells were incubated with Alexa-Fluor-594-conjugated anti-mouse-IgG (MOLECULAR PROBES, MA, USA). Changes in cell proliferation were calculated from the absorbance readings at 370 nm (490 nm reference wavelength) on the Benchmark Plus microplate reader (BIO-RAD, Australia).

### Colony formation

For the colony-formation assay, 250 MelJD and MelCV cells were seeded in 6-cm^2^ plates and kept under WFA treatment for 72 h, and grown for 10 days to allow colonies to form. Colonies were fixed and stained with crystal violet solution (SIGMA) then washed with water to remove unincorporated stain. Cells were photographed and colony formation was quantified from crystal violet absorbance readings at 590 nm on the Benchmark Plus microplate reader (BIO-RAD).

### Determination of cell death

Mitochondrial membrane potential of cells was determined using a MITOPROBE DIIC_1_(5) Assay Kit (LIFE TECHNOLOGIES). 2 × 10^4^ melanoma cells per well and 8 × 10^3^ fibroblast cells were cultured in 96-well plates and treated with WFA for 48 h. After treatment, cells were incubated with DILC_1_(5) working solution (50 nM/well) for 30 min, and the fluorescence of DILC_1_(5) was measured at 630-nm excitation and 670-nm emission wavelengths using VICTOR Multilabel Counter (PERKINELMER).

In addition, apoptosis was also assessed by measuring phosphatidylserine translocation with FITC-conjugated Annexin-V using cell apoptosis kit I (BD BIOSCIENCE, San Jose, CA, USA). Briefly, MelJD and MelCV cells were treated with increasing concentrations of WFA (0–5 µM), then double stained with 7AAD and Annexin-V-FITC, following the manufacturer’s protocol. Untreated cells with no stain were used as a negative control. Fluorescence intensity was measured by the FACS-CANTO Flow Cytometer (BD BIOSCIENCE).

The cytotoxic effects of WFA treatment were determined by SYTOX Green staining (LIFE TECHNOLOGIES). Melanoma cells (2 × 10^4^ cells per well) were cultured in 96-well plates and treated with WFA for 48 h. Supernatants were then discarded, and the cells were incubated with SYTOX Green working solution (30 nM/well) for 30 min. The fluorescence of SYTOX Green was measured at 490-nm excitation and 520-nm emission wavelengths using VICTOR Multilabel counter (PERKINELMER).

### Cell migration assay

MelJD and MelCV cells were serum starved prior to seeding into a trans-well insert (BD BIOSCIENCES) along with WFA. Trans-well inserts were placed in a companion plate with 5% FCS media as chemo-attractant, plates were then incubated for an additional 18 h. After incubation, cells were fixed with methanol, and stained with May-Grunwald (SIGMA-ALDIRCH, Australia) and Giemsa Stain (SIGMA-ALDRICH). Data was generated by counting cells under microscope (OLYMPUS, × 20 objective). Migration index was calculated by dividing migrated cell with total number of cells in wells.

### Western blot analysis

Cell pellets were lysed with Radioimmunoprecipitation assay (RIPA) buffer freshly supplemented with protease inhibitor cocktail (SIGMA-ALDRICH). Protein lysate was standardized using the BCA protein quantitation assay kit as per manufacturer's instructions (THERMO SCIENTIFIC, IL, USA)*,* and 20–40 µg whole protein lysates were resolved on either 10.5% or 10–14% Tris–HCl Criterion gels (BIO-RAD, Gladesville, NSW, Australia). Nitrocellulose membranes (GE HEALTHCARE, Rydalmere, NSW, Australia) were blocked with 5% (wt/vol) nonfat dry milk in Tris-buffered saline with Tween-20 (20 mM Tris–HCl (pH 7.6), 137 mM NaCl, 0.1% Tween-20), then incubated overnight at 4 °C with the following primary antibodies: TRIM16 (1:1000; BETHYL LABORATORIES, TX, USA) and GAPDH antibody (1:1000; SANTA CRUZ BIOTECHNOLOGIES, Texas, USA). Appropriate horseradish peroxidase conjugated secondary antibodies (1:3000; SANTA CRUZ BIOTECHNOLOGIES and MERCK MILLIPORE, Australia) were diluted in Tris-buffered saline with 0.1% Tween-20 and membranes were probed on room temperature for 2 h. Immunoblots were visualized with SUPER SIGNAL WEST PICO Chemiluminescence reagents (THERMO SCIENTIFIC PIERCE, Australia). Densitometry of protein expression was measured using Image Lab software (https://www.bio-rad.com/en-au/product/image-lab-software?ID=KRE6P5E8Z) (BIO-RAD) and each protein expression band was normalized to GAPDH loading control.

### RNA isolation and quantitative real-time PCR (RT-qPCR)

Total RNA was extracted using the PURELINK RNA kit (LIFE TECHNOLOGIES) according to the manufacturer's protocol. 1 μg total RNA was reverse-transcribed using the TETRO cDNA synthesis kit (BIOLINE, Australia). 1 μL of purified cDNA (0.1–1 μg) was added to a reaction mix containing, 2.5 μL 10× PCR buffer, 1.5 μL 25 mM MgCl, 2.5 μL of 10 mM dNTPs, 0.5 μL GOLD TAQ (INVITROGEN) and 1 μL of each of forward and reverse TRIM 16 primers (10 nM): Forward CAGGCTCCAGGCTAACCAAAAG and Reverse TCCTCTAAGAAGGGCATCACATTG. Gene expression was verified using Power SYBR GREEN MIX (APPLIED BIOSYSTEMS, LIFE TECHNOLOGIES) PERFORMED ON ABI7500 THERMO-CYCLER (APPLIED BIOSYSTEMS, LIFE TECHNOLOGIES) with a standard protocol. Differential gene expression was measured using the log2∆∆Ct analysis. All mRNA expression levels were normalized to glyceraldehyde-3-phosphate dehydrogenase (GAPDH).

### Statistical analysis

Data were analysed with Prism 7 software (GRAPHPAD) and results are presented as the mean ± SEM. All statistics were based on continuous variables. For single comparisons, differences were determined by using a two-tailed, unpaired Student's *t*-test with a confidence interval (CI) of 95%. For multiple comparison one-way ANOVA was used. *P* ≤ 0.05 was denoted as statistically significant. Drug dose–response curves were analysed with a nonlinear regression curve fit model. The p values are as indicated on images. Analyses were not performed in a blinded manner.

### Ethical approval

This article does not contain any studies with human participants or animals performed by any of the authors.

### Informed consent

For this type of study, formal consent is not required.

## Supplementary information

Supplementary information.

## Data Availability

All data generated or analyzed during this study are included in this published article. Uncropped and unprocessed immunoblot scans for all main figure immunoblots are provided as Supplementary Information.
