# Supplementary material for: Withaferin A activates TRIM16 for its anti-cancer activity in melanoma
Source: Sci Rep. 2020 Nov 12;10:19724. doi: 10.1038/s41598-020-76722-x (PMC7665169; doi:10.1038/s41598-020-76722-x)
Supplement: Supplementary file 1 — Supplementary information. [file 41598_2020_76722_MOESM1_ESM.pdf]

Withaferin A activates TRIM16 for its anti-cancer activity in melanoma

Zsuzsanna Nagy[1,2,*], Belamy B. Cheung[1,2,3,*,#], Wing Tsang[1], Owen Tan[1], Mika Herath[1], Olivia C. Ciampa[1], Fatima Shadma[1], Daniel R. Carter[1,2,4] and Glenn M. Marshall[1,5,#]

[1] Children's Cancer Institute Australia for Medical Research, Lowy Cancer Research Centre, UNSW Sydney, Australia

[2] School of Women's & Children's Health, UNSW Sydney, Randwick NSW 2031, Australia

[3] Academy of Medical Sciences, Zhengzhou University, Henan, China

[4] School of Biomedical Engineering, University of Technology, Sydney, NSW 2007, Australia

[5] Kids Cancer Centre, Sydney Children's Hospital, Randwick 2031, NSW, Australia

[*] Joint first authors

#Corresponding Authors: Belamy B. Cheung, Children's Cancer Institute Australia, UNSW Sydney, PO Box 81, Randwick NSW 2031, Australia. Phone: 61(02) 9385 2450, Fax: 61 (02) 9662 6584, E-mail: bcheung@ccia.unsw.edu.au or Glenn M. Marshall, Sydney Children's Hospital, Level 1, South Wing, High Street, Randwick 2031, NSW Australia. Phone: 61 (02) 9382 1721, Fax: (02) 9382 1789, E-mail: g.marshall@unsw.edu.au

Supplementary Figure Legends

Supplementary Fig. 1.

Representative figures of flow cytometry analysis in vehicle control, 1 µM and 5 µM WFA treated MelJD and MelCV cells. The bottom left quadrant represents living cells, the cells in top left quadrant represents necrotic, the top right quadrant represents late apoptosis and the bottom right quadrant represents cells undergoing early apoptosis.

Supplementary Fig. 2.

Densitometry analysis of TRIM16 expression in MelJD and MelCV cells following TRIM16 siRNAs knockdown. GAPDH was used as internal control.

Supplementary Fig. 3.

(A) The full-length blots of the Figure 3A

(B) The full-length blots of the Figure 4A

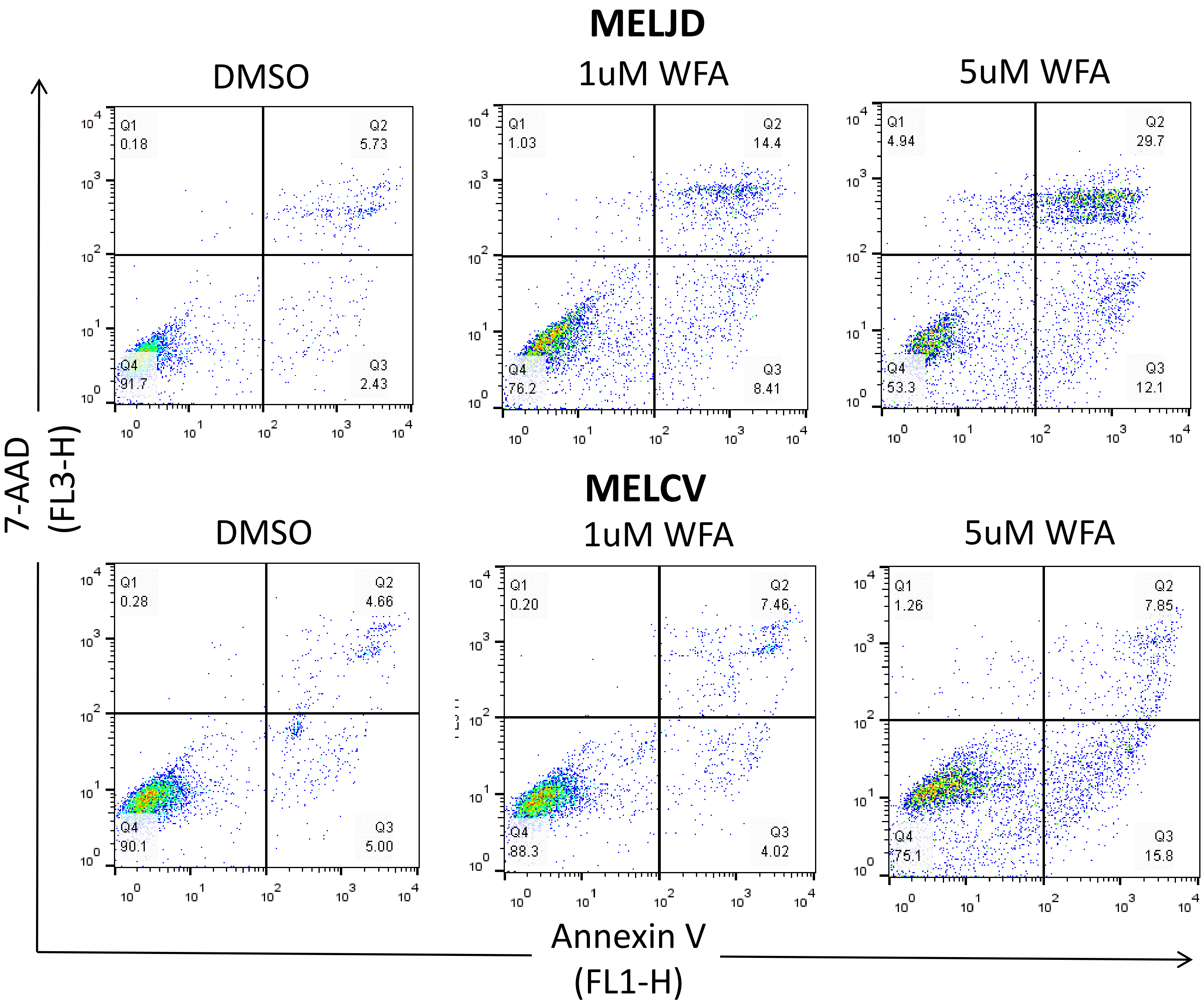

Supplementary Figure 1

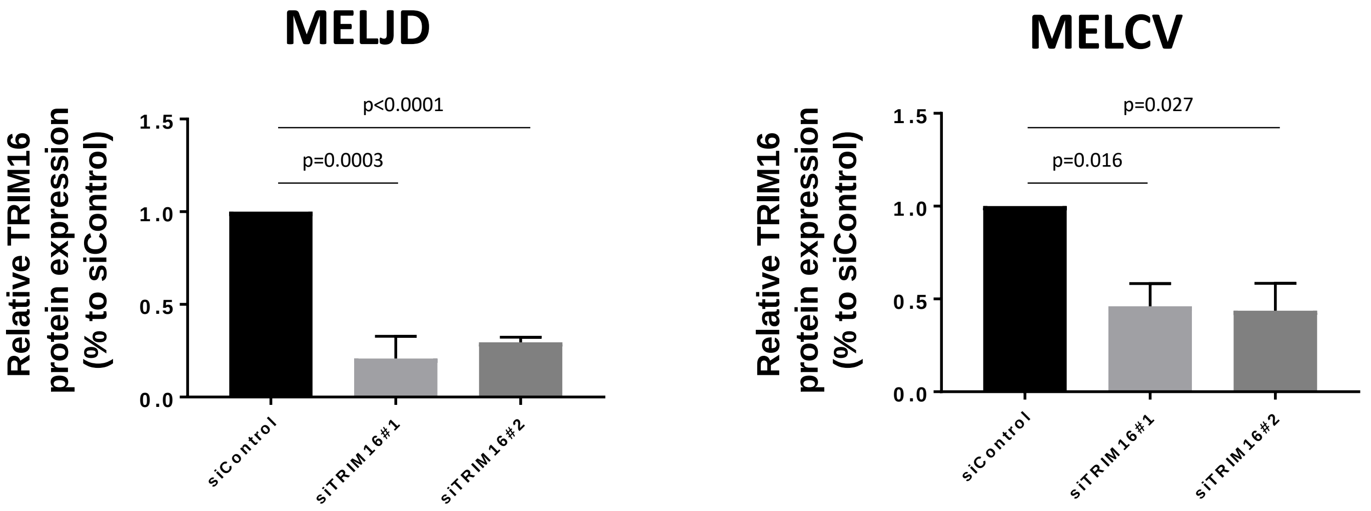

Supplementary Figure 2

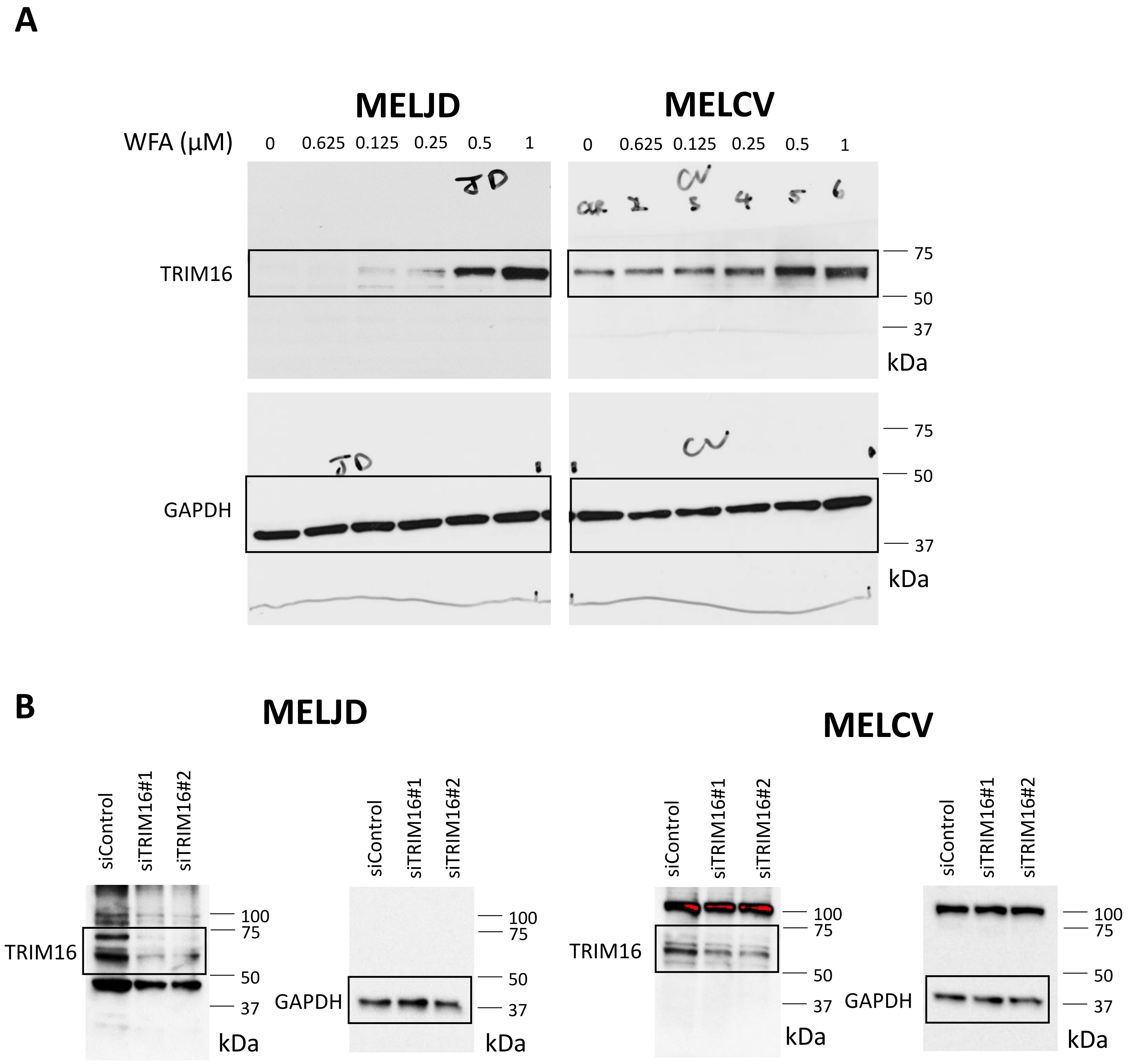

Supplementary Figure 3
